# Short term *ex-vivo* expansion of circulating head and neck tumour cells

**DOI:** 10.18632/oncotarget.11159

**Published:** 2016-08-09

**Authors:** Arutha Kulasinghe, Chris Perry, Majid E. Warkiani, Tony Blick, Anthony Davies, Ken O'Byrne, Erik W. Thompson, Colleen C. Nelson, Ian Vela, Chamindie Punyadeera

**Affiliations:** ^1^ The School of Biomedical Sciences, Institute of Health and Biomedical Innovation, Queensland University of Technology, Kelvin Grove, QLD, Australia; ^2^ Department of Otolaryngology, Princess Alexandra Hospital, Woolloongabba, QLD, Australia; ^3^ School of Mechanical and Manufacturing Engineering, Australian Centre for NanoMedicine, University of New South Wales, Sydney, Australia; ^4^ Translational Cell Imaging Queensland, Institute of Health and Biomedical Innovation, Queensland University of Technology, QLD, Australia; ^5^ Australian Prostate Cancer Research Centre-Queensland, Institute of Health and Biomedical Innovation, Queensland University of Technology, Princess Alexandra Hospital, QLD, Australia; ^6^ Department of Urology, Princess Alexandra Hospital, Wolloongabba, QLD, Australia; ^7^ Translational Research Institute, Woolloongabba, QLD, Australia

**Keywords:** circulating tumour cells, head and neck cancer, ex-vivo culture, metastasis, HPV

## Abstract

Minimally invasive techniques are required for the identification of head and neck cancer (HNC) patients who are at an increased risk of metastasis, or are not responding to therapy. An approach utilised in other solid cancers is the identification and enumeration of circulating tumour cells (CTCs) in the peripheral blood of patients. Low numbers of CTCs has been a limiting factor in the HNC field to date. Here we present a methodology to expand HNC patient derived CTCs *ex-vivo*. As a proof of principle study, 25 advanced stage HNC patient bloods were enriched for circulating tumour cells through negative selection and cultured in 2D and 3D culture environments under hypoxic conditions (2% O_2_, 5% CO_2_). CTCs were detected in 14/25 (56%) of patients (ranging from 1–15 CTCs/5 mL blood). Short term CTC cultures were successfully generated in 7/25 advanced stage HNC patients (5/7 of these cultures were from HPV+ patients). Blood samples from which CTC culture was successful had higher CTC counts (*p* = 0.0002), and were predominantly from HPV+ patients (*p* = 0.007). This is, to our knowledge, the first pilot study to culture HNC CTCs *ex-vivo*. Further studies are warranted to determine the use of short term expansion in HNC and the role of HPV in promoting culture success.

## INTRODUCTION

Head and neck cancers are the seventh most common cancer globally. Approximately 50% of the patients die within 5 years. Current diagnostic methods include clinical assessment, imaging and tissue biopsy [1]. When metastases are clinically evident, treatment is palliative in nature only. There are currently no methods to predict which patients with a higher disease burden will develop metastases. The ability to do so would potentially lend itself to treatment escalation at diagnosis. Circulating tumour cells (CTCs) found in the lymphovasculature are thought to have the propensity to metastasize at distant sites. CTCs are extremely rare events (typically a few CTCs per 10^6–9^ blood cells), therefore very sensitive methodologies are required to isolate these cells [2]. Enrichment strategies aim to increase the CTC concentration from blood by several log units for easier detection by downstream methodologies. Biological approaches for CTC enrichment can include positive selection using anti-epithelial marker antibodies (EpCAM), anti-mesenchymal marker antibodies (N-cadherin) or negative selection using antibodies against CD45 to deplete leukocytes [3]. Moreover, the physical properties of CTCs can be employed for cell separation (size, density, electric charge and deformability [3–5].

There has been a shift in CTC enrichment and detection technologies away from positive selection marker based platforms such as the FDA-approved CellSearch^®^, to label free methodologies [6]. CellSearch^®^ CTC enumeration has shown clinical relevance in EpCAM+ CTCs across multiple tumour types, however, to date, none of the other technologies have been clinically validated [7]. Currently, the CIRCUTEC clinical trial is investigating CTCs as an early predictive marker or response to a first line anti-EGFR therapy (Cetuximab) in metastatic head and neck squamous cell carcinoma. During enumeration by CellSearch^®^, cells are fixed before isolation, disallowing propagation. Technologies that are able to keep CTCs viable are desirable in the field, especially non-marker based assays that utilise negative selection techniques capable of capturing a larger CTC population from the patient's circulation.

One way to enable the molecular and functional analysis of the few CTCs that are present in circulation is to expand the CTCs *ex-vivo*. This has shown promise in breast, prostate and lung cancer where short term CTC culture has been performed [4, 8, 9]. Until recently, CTC expansion *ex-vivo* was the bottleneck in the field. The need for new isolation technologies that preserve the CTCs integrity in order to perform functional analysis has been a recent emphasis [9, 10]. Viable CTCs grown in culture can potentially be used for therapeutic screening and used to guide patient therapy. Studies have shown in small-cell lung cancer that CTC-derived explants mirror the donor patient's response to chemotherapeutics [11]. Through the creation of CTC cell lines and analysis, personalized drug screening can be performed revealing the mutational landscape and identifying new therapeutic targets [12].

Recently, by use of a novel 3D organoid system, the long term culture of prostate cancer from patient biopsies and CTCs using a defined media was reported [8]. In this pilot study, we aimed to use the defined media, to culture HNC patient derived CTCs in 2D and 3D formats.

## RESULTS

### Patient characteristics

A total of 25 HNC patients were recruited for this study and for all samples CTC cultures attempted. The median age was 60 years (range 49–85); men (*n* = 20), women (*n* = 5). All the patients were at advanced clinical stage ranging from T_3_N_0_ to T_4a_N_2b_. Distant metastasis was assessed by an MDT clinic using advanced imaging platforms. HNC patients (100%) were found to be radiographically M_0_ and 28% were HPV-positive. The clinical details of the short term culture positive samples is presented on Table 1.

**Table 1:** Clinical details, CTC counts and culture notes of short term culture positive samples

Patient #	Tumour Location	Staging	HPV status	CTC count /5ml blood	Culturability
1	Oropharynx	T4aN2a	+	4	20% confluency on day 14 Proliferative up to day 21 in 2D
6	Oropharynx	T4N0	+	3	10% Confluency on day 14 Proliferative up to day 21 in 2D
7	Nasopharynx	T4N0	+	3	20% confluency on day 14 Proliferative up to day 21 in 2D
9	Oropharynx	T4aN2b	–	7	60% confluency on day 14 Proliferative up to day 25 in 2D and 50 days in 3D
10	Oropharynx	T3N1aM0	+	15	70% confluency on day 14 Proliferative up to day 50 in 2D and 63 days in 3D
14	Oropharynx	T4aN2b	+	4	40% confluency on day 14 Proliferative up to day 28 in 2D
17	Supraglottis	T3N0	–	9	70% confluency on day 14 Proliferative up to day 25 in 2D and Day 40 in 3D

### RosetteSep™

**Figure 1:**
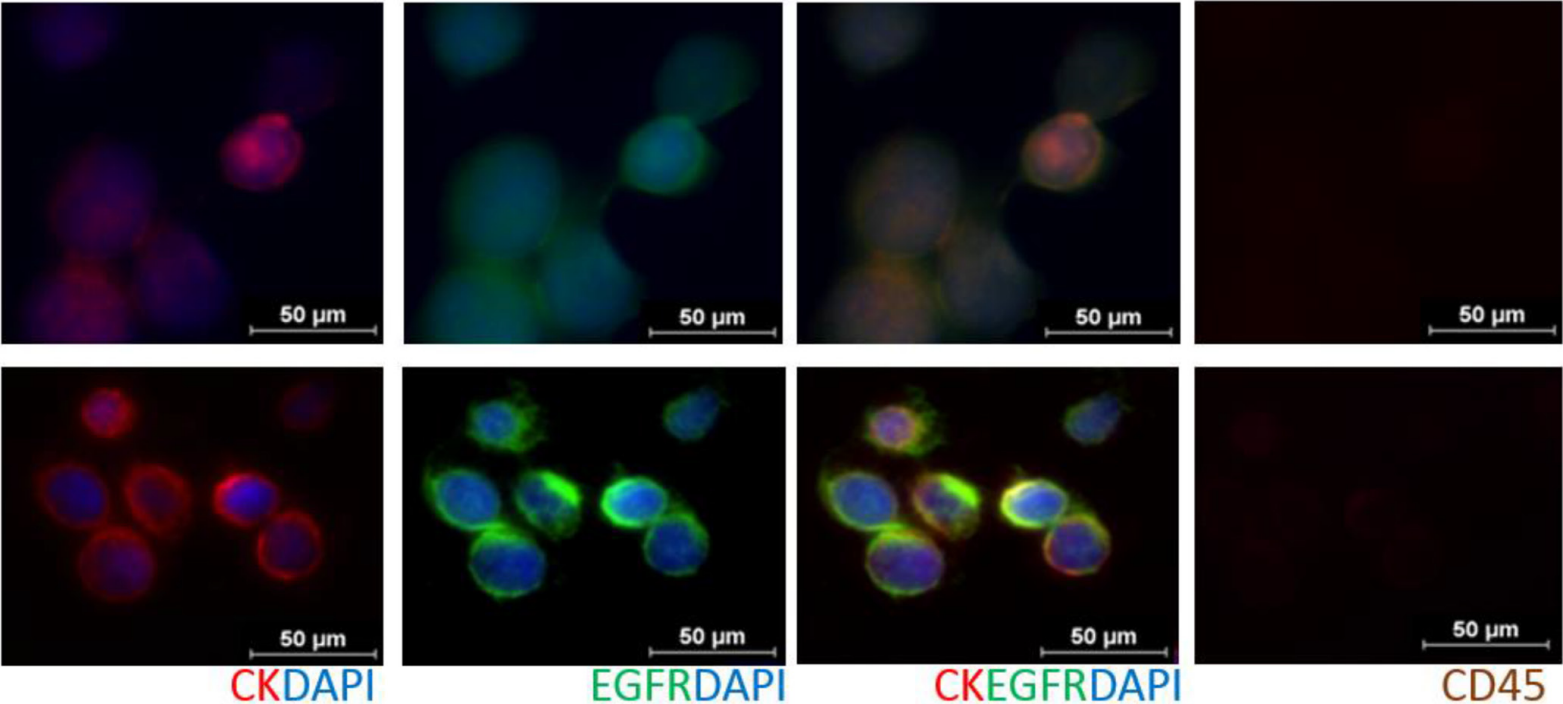
Immunofluorescent staining of CTCs from RosetteSep™ enrichred head and neck cancer patient blood samples (Row 1: Pt #10, Row 2: Pt #17), stained with an antibody cocktail (Cellsearch^®^: anti-Cytokeratin 8/18/19, DAPI, anti-CD45) and anti-EGFR Cells found to be CD45 negative.

The RosetteSep™ protocol was used to enrich for CTCs in all 25 HNC patient samples. CTCs were detectable 14/25 (56%) of the samples (Figure 1). It should be noted that only half of the enriched volume was cytospun onto a glass slide to be stained and the other half was put into culture. This ensured a CTC count at time point zero from the equivalent of 5 mL of whole blood.

### CTC *ex-vivo* expansion

**Figure 2:**
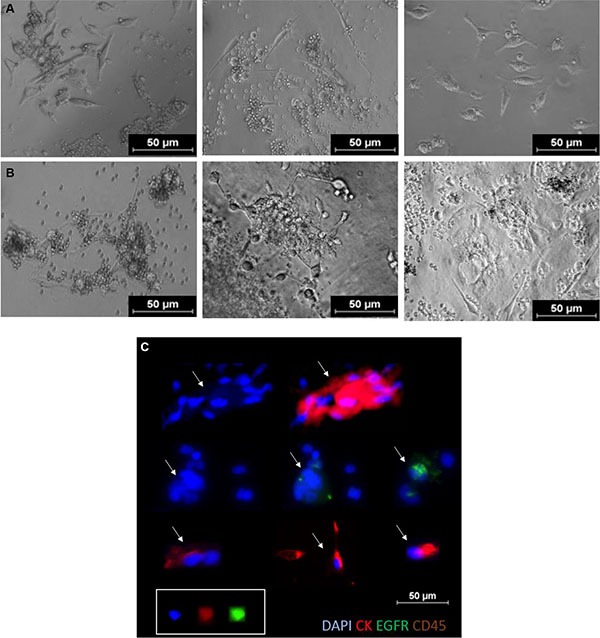
Short term CTC cultures in 2D (MSK media, day 7, 2% O_2_) Row (**A**) 96 well standard microplate, Row (**B**) Corning^®^ Spheroid Microplates (ThermoScientific, USA). (**C**) In well staining of proliferating cells in culture at day 7. Immunofluorescent staining with combination antibody (Cellsearch™: Cytokeratin 8,18–19, DAPI, CD45) and EGFR. These cells were found to be CD45 negative (not shown). Arrows represent single/clusters of CTCs.

**Figure 3:**
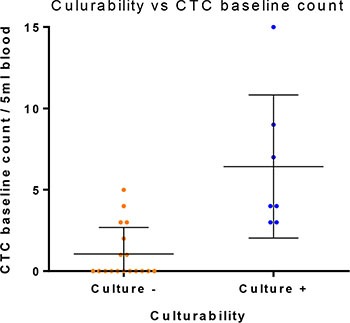
CTC count vs short term culture success *P* = 0.0002; Mann-Whitney test. Error bars represent standard deviation.

**Figure 4:**
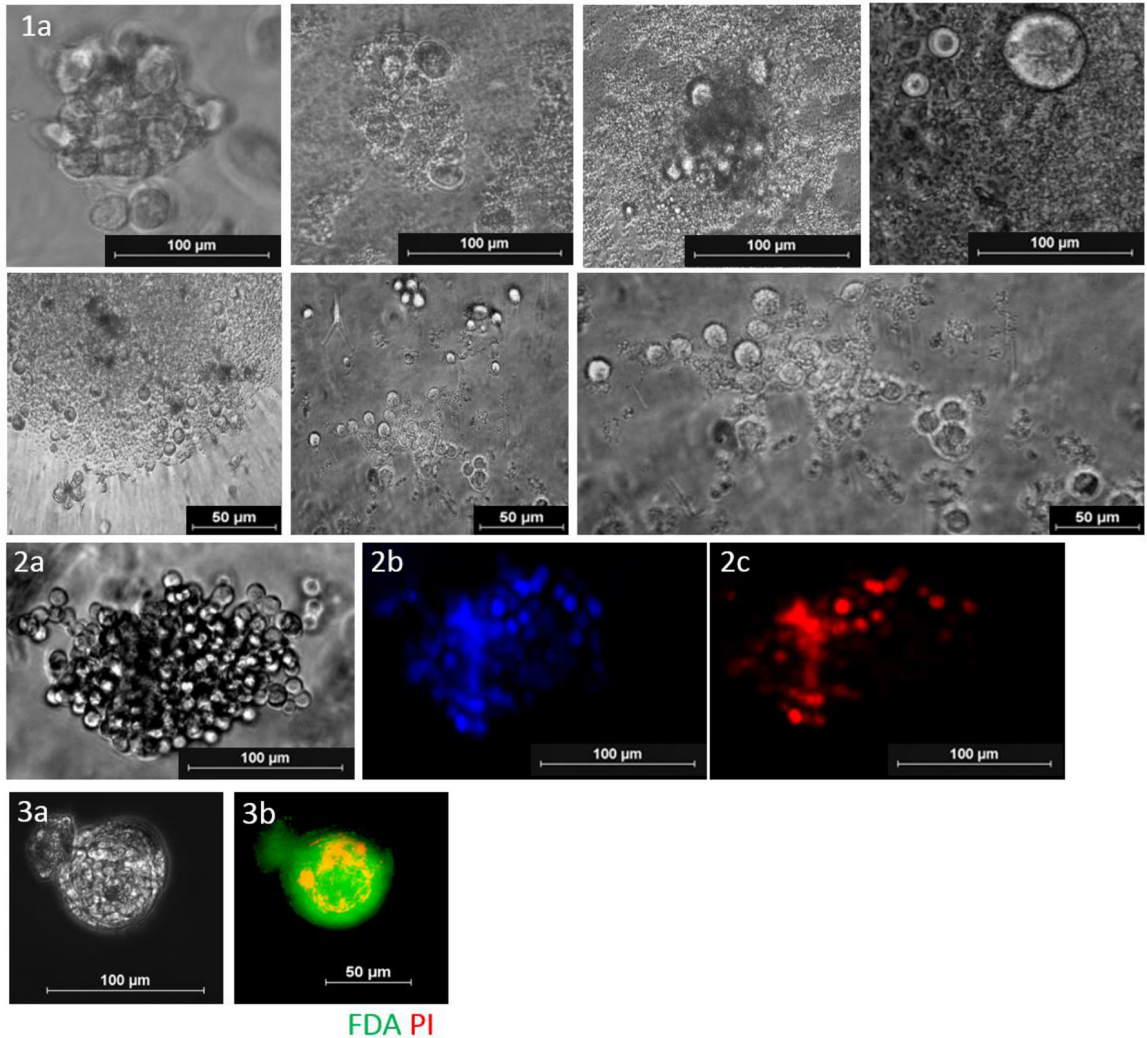
(**1A**) Short term CTC cultures in 3D (MSK media, day 14, 2% O_2_). (**2A**) Cell cluster under bright field. (**2B**) CellSearch^®^ DAPI immunofluorescent staining (**2C**) CellSearch pan-cytokeratin immunofluorescent staining. These cells were found to be CD45 negative (**3A**) Spheroid under bright field (**3B**) Live/dead immunofluorescent stain (FDA; green) (PI; red) showing a necrotic core.

Short term cultures were successful propagated in 7/25 samples (Table 1 and Figure 2). All patients presenting with successful cultures had CTCs detectable at time point zero by immunofluorescent staining (Figure 1). 5/7 of the expandable samples had patient primary HNCs originating from the oropharyngeal region. Blood samples from which CTC culture was successful had higher CTC counts (Figure 3: *p* = 0.0002; Mann-Whitney test), as were those from HPV+ patients (Table 2: *p* = 0.007; Fisher's exact test). In patients 1, 6 and 7 (< 5CTCs/5 ml blood), a low CTC cell confluency was reached in 14 days, which continued till Day 21 (Supplementary Figure S2). In patients 9 and 10 (> 7CTC/5 ml blood), CTCs were highly confluent on day 14, proliferative up to day 35 and day 50 in 2D respectively, and 2 weeks longer in the Happy Cell^®^ (Supplementary Figure S3). In patient 14 (4 CTCs/5 ml blood), 40% confluency was reached at day 14 and remained proliferative till day 28. In patient 17 (9 CTCs/5 ml blood), 70% confluency was reached on day 14, remained proliferative till day 25 in 2D and day 40 in 3D. In all patients with a CTC count of more than 5CTCs/5 mL blood, the cell confluency on day 14 was much higher each time and remained viable for longer than when less than 5 CTCs were found. In cultured spheroids, a necrotic core was found in most cases (Figure 4). As a control, 4 normal healthy volunteers were screened and no CTC-events were observed in 2D/3D culture (Supplementary Figure S1).

**Table 2:** Correlation between HPV status and short term culture success

	Culture negative	Culture positive
HPV-negative	16	2
HPV-positive	2	5

*P* = 0.007; Fisher's exact test.

## DISCUSSION

CTCs were detected in 14/25 of the advanced stage HNC patients ranging from 1–15 CTCs/5 mL patient blood. In the 10 mL blood draw, the actual CTC count in these late stage patients could be approximated to be higher. The number of CTCs was determined at time point zero to establish a CTC count prior to culture. By use of a label-free technology, we are able to show that higher CTC counts are found than previously reported for CTC studies in HNC utilising the EpCAM biased Cellsearch^®^ system [1, 13, 14]. However, the discovery and validation of new CTC markers remains in its infancy [3, 15].

The short term cultures in this study were from advanced stage HNC patients, the bulk of which were HPV positive (71.4%) and originating from the oropharynx. HPV oncogenes E6/E7 have been shown to inactivate tumour suppressor genes p53 and Rb, inducing a replicative senescent state for the immortalization of primary cells. In the first 3 patients (#1,6–7), CTCs were successfully cultured up to 21 days under low cell confluency (10–20%). There were sporadic areas of growth in 2D with high cell populations in those select areas reflecting cells which may have proliferated more rapidly. This highlighted that a high initial CTC density was required to promote *ex-vivo* culture [9]. In patients (#9,10,17), with higher CTC counts at time zero, much higher confluences were reached at day 14 (60–70%), reflecting the higher initial CTC numbers. These patient CTCs were cultured in 3D, and in all 3 cases remained more viable than in 2D. A high CTC count has been a requirement for long term patient cultures and the creation of stable cell lines reflecting the high CTC density required for *ex-vivo* propagation [8, 9, 16, 17]. 2D and 3D culture formats tended to proliferate better in standard 96-well microplates and spheroid microplates respectively.

This study demonstrates that HNC patient derived CTCs can be expanded *ex-vivo*. The importance of *ex- vivo* CTC work has been demonstrated in numerous other cancer models [16–19]. Further studies are warranted in the HNC field that create cell lines which can be used to test new therapeutic approaches. More importantly, novel immunotherapies are being developed against proteins that can block the immune response of T-cells such as PD-L1 [19], which has shown to be in frequently expressed in breast cancer [10]. For the establishment of CTC cell lines in HNC patients, isolation of higher numbers of CTCs is warranted. This is currently possible through leukopheresis where litres of blood are filtered for CTCs rather than the conventional 10 mL [20]. Alternatively identifying HNC patients with a heavy burden of disease and known metastatic disease may provide higher CTC counts for enrichment and culture. However, the clinical importance is in extending this technology to early stage patients. Low-risk patients presenting with CTCs could be recruited into clinical trials to determine the benefit of adjuvant therapy as opposed to waiting for overt metastasis to develop [21].

## MATERIALS AND METHODS

### Patient cohort

Ethics approval was obtained by the Metro South Health Service District Research Ethics committee in accordance with the National Health and Medical Research Council's guidelines (HREC/12/QPAH/381) to collect blood from the Princess Alexandra Hospital (PAH). This study has UQ ethics approval (2009000779) and QUT ethics approval (1400000617). After written informed consent was obtained from the participants, 10 mL blood samples were obtained from patients presenting with HNC. The clinicopathological patient findings are presented in Table 3.

**Table 3:** Patient demographics

Variables	*N*
Total	25 (100%)
Gender	
Male	20 (80%)
Female	5 (20%)
***Age, y***	
< 60	10 (40%)
> 60	15 (60%)
***Anatomic site of primary***	
Oral Cavity	6 (24%)
Oropharyngeal	12 (48%)
Supraglottis	3 (12%)
Hypophayngeal	1 (4%)
Larynx	1 (4%)
Nasopharyngeal	2 (8%)
***Tumour Size***	
T1	0
T2	0
T3	8 (32%)
T4	17 (68%)
***Nodal spread***	
N0	5 (20%)
N1	2 (8%)
N2a-c	18 (72%)
***Distant metastases***	
M0	25 (100%)
M1	0
***HPV status***	
HPV-positive	7 (28%)
HPV-negative	18 (72%)
***CTC status***	
CTC-positive	14 (Range from 1–15 CTCs/5 ml whole blood)
CTC-negative	11

### Preparation of blood samples and CTC enrichment

Whole blood (10 mL) was collected in heparin tubes (BD-Plymouth, UK) and incubated with RosetteSep™, Human CD45 depletion cocktail (Stemcell Technologies™, Vancouver, Canada) at 50 μl/mL of whole blood for 20 minutes at room temperature. Cellular separation was performed in SepMate™-50 mL tubes containing a density gradient medium - Lymphoprep™ (Stemcell Technologies™, Vancouver, Canada). Half of the cell suspension was spun onto glass slides using the Cytospin™ 4 Cytocentrifuge (ThermoScientific, USA), air dried and fixed with 4% PFA. The presence of CTCs was verified by immunofluorescent staining and analysed using an Olympus IX3 Inverted microscope. The other half of the enriched cellular suspension was cultured in 2D and 3D media under hypoxic conditions.

### CTC parameters

CTCs were visualized using immunofluorescence post RosetteSep™ enrichment and in culture using the Cellsearch^®^ (Janssen Diagnostics) antibody stain (Cytokeratin 8, 18, 19, CD45 and DAPI). Cells were classified as CTCs if (i) Morphologically larger than the background cells with intact nuclei (ii) High nucleus-cytoplasmic ratio (iii) pan-cytokeratin positive (iv) CD45 negative. Further characterization was done using (v) EGFR immunostaining (AY13) (Biolegend, CA, USA). and DNA FISH (Supplementary Figure S4) Stained slides were manually examined by two trained users and questionable events evaluated until consensus was reached. The results were reported as number of CTCs/5 mL whole blood.

### *Ex-vivo* expansion of CTCs

Isolated CTCs were cultured in the following formats: 96 well standard microplates, 96F non adherent microplates, spheroid microplates (Thermo Scientific, USA) and specialized microwell formats with a culture medium containing Advanced DMEM/F12 with the following additives: 50 ng/mL EGF (Sigma), 5% v/v R-spondin 1, 10% v/v Noggin, 10 ng/mL FGF10 (Peprotech), 1 ng/ml FGF2 (Peprotech), 10 nM Nicotinamide (Acros), 0.5 μM A83–01 (Tocris), 10 μM SB202190 (Sigma Aldrich), 10 μM Y-27632 (Selleck Chemical), 1X B27 Additive (Invitrogen), 1.25 mM N-Acetyl-L-cysteine (Sigma-Aldrich), 2 nM Glutamax (Invitrogen), 10 mM HEPES (Sigma Aldrich), 1:100 v/v Primocin (Invivogen). For 3D *ex-vivo* expansion, Happy Cell^®^ (Biocroi Ltd, Dublin, Ireland) hydrogel was used in combination with the above mentioned cocktail to provide the environment for the CTCs. To perform spheroid staining, an inactivation buffer was added to the culture for 1 hour at 37^o^C to collapse the Happy Cell^®^ matrix. Cultures were incubated under hypoxic conditions (2% O_2_, 5% CO_2_) and imaged weekly starting on day 7.

### Live/dead staining

Spheroids found in cultures were simultaneously stained with two fluorescent dyes to distinguish between live and dead cells as per manufacturer's instruction. Fluorescein diacetate (FDA) (Thermoscientific, USA) stains live cells green by converting into green fluorescent metabolite fluorescein and propidium iodide (PI) stains dead cells red.

### Hypoxia

A number of *ex-vivo* culture conditions were tested to identify the optimal conditions that were used in this study. Initially 8% oxygen was used for CTC culture and CK^+^DAPI^+^ cells were observable at day 14, although the cells appeared to remain dormant until this time point. When the oxygen tension was brought down to 2%, these CK^+^DAPI^+^ cells were observable at day 7. The lower oxygen tension was preferred as this resulted in the proliferating cells at an earlier time point, which clinically meant a faster turnaround time.

### Statistical analysis

The association between initial CTC counts and short term culture success was evaluated using the Mann-Whitney test. The association between the clinicopathological factors and short term culture success was evaluated using the Fisher's exact test. *P* values less than 0.05 were considered statistically significant.

## CONCLUSIONS

In this proof of principle study, we have shown that HNC patient derived CTCs can be cultured short term. Having said this, the underlying mechanisms involved such as co-infection with HPV need to be investigated. These findings need to be validated in larger cohorts of patients.

## SUPPLEMENTARY MATERIALS FIGURES


