# Supplementary material for: Short term ex-vivo expansion of circulating head and neck tumour cells
Source: Oncotarget. 2016 Aug 9;7(37):60101–9. doi: 10.18632/oncotarget.11159 (PMC5312371; doi:10.18632/oncotarget.11159)
Supplement: Supplementary file 1 [file oncotarget-07-60101-s001.pdf]

Short term *ex-vivo* expansion of circulating head and neck tumour cells

Supplementary Materials

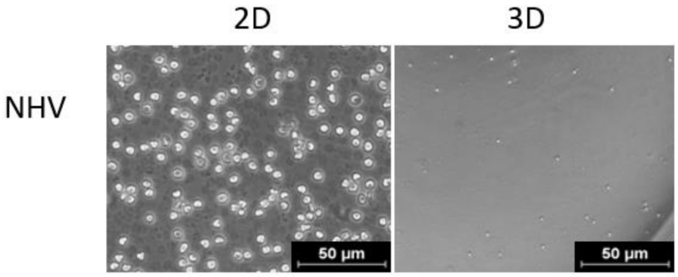

Supplementary Figure S1: Normal healthy volunteer samples enriched by RosetteSep, cultured in the MSK media in 2D and 3D formats. No CTC-like events were observed in culture over 56 days.

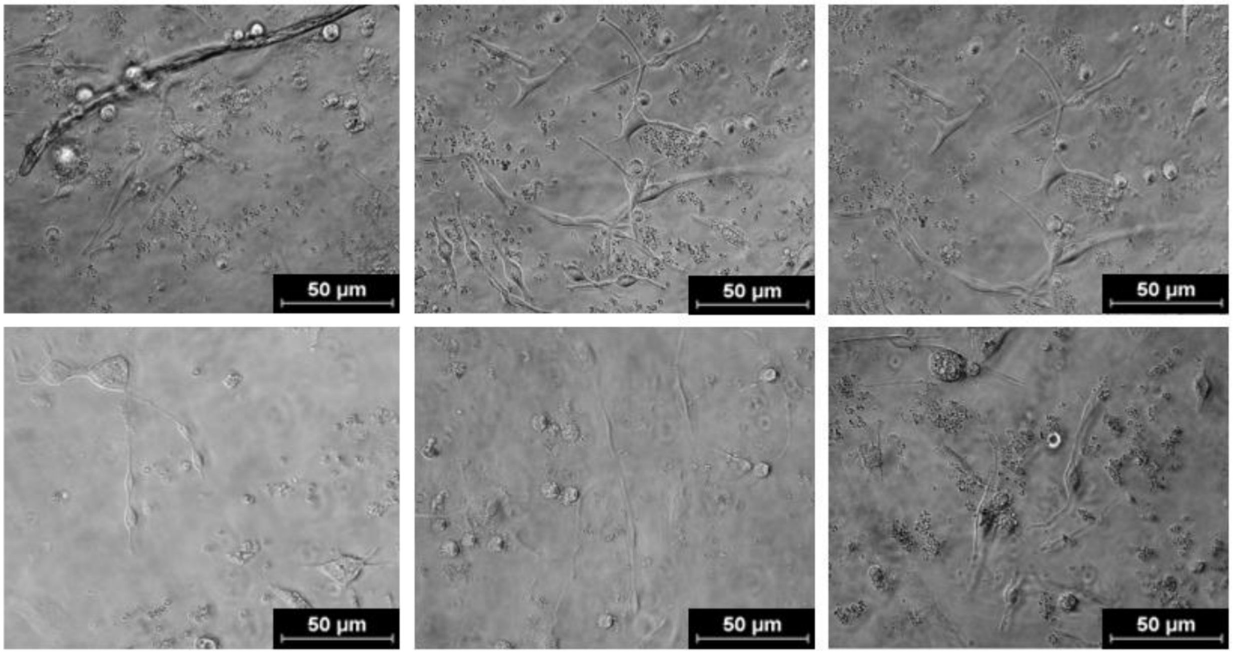

Supplementary Figure S2: Short term CTC cultures in 2D (MSK media, day 21, 2% O^2) in 96 well standard microplate (ThermoScientific). Patient number 7 and 1.

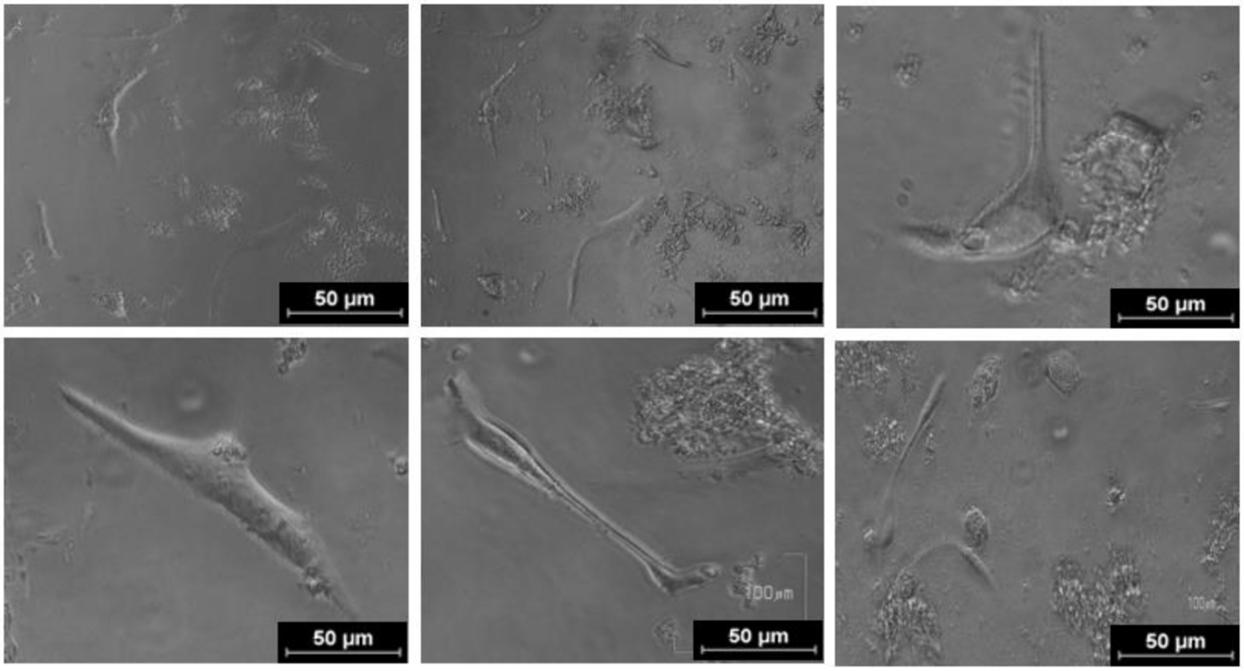

Supplementary Figure S3: Short term CTC cultures in 3D (MSK media, day 56, 2% O^2) in 96 well standard microplate (ThermoScientific). Patient number 1.

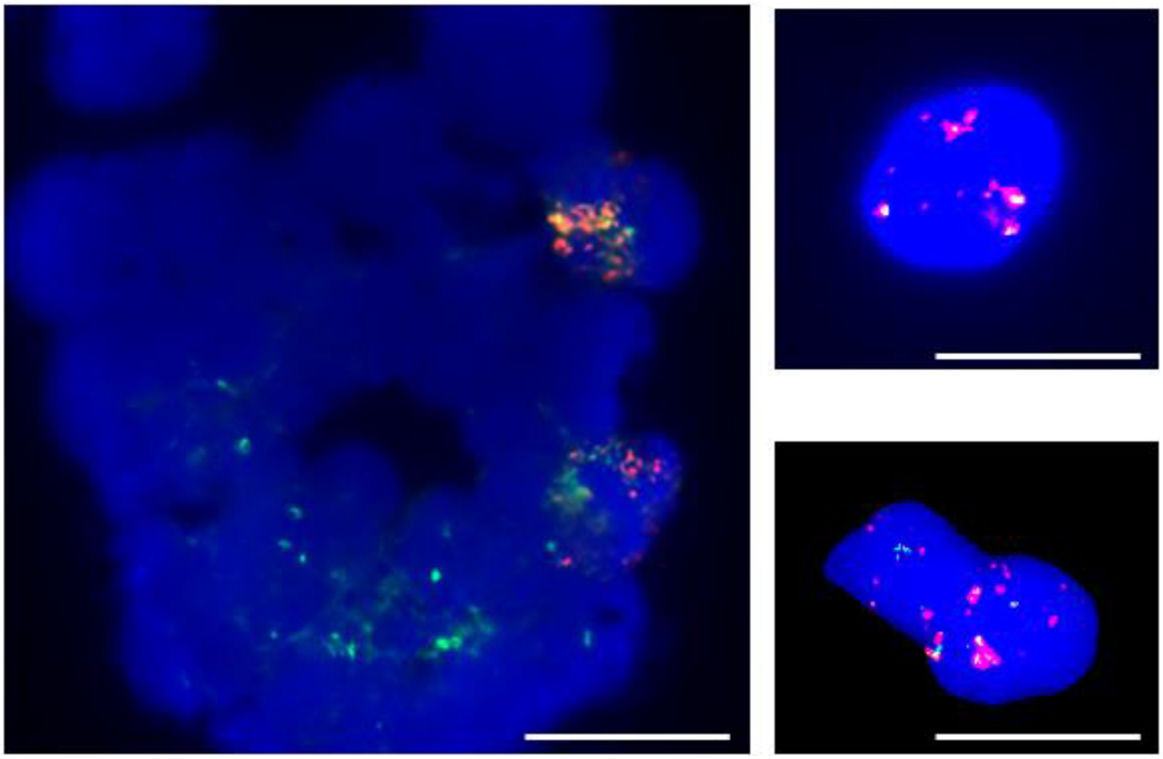

Supplementary Figure S4: Merged images (DAPI, spectrum green, spectrum red) of DNA FISH processed CTCs using EGFR target probe (red) in each patient sample, demonstrating EGFR amplification in contrast to centromere for chromosome 7 (CEN7, green). Scale bar, 10 µm.
